# Deltex3 inhibits Epithelial Mesenchymal Transition in Papillary Thyroid Carcinoma via promoting ubiquitination of XRCC5 to regulate the AKT signal pathway

**DOI:** 10.7150/jca.48141

**Published:** 2021-01-01

**Authors:** Lidong Wang, Yonglian Huang, Chenxi Liu, Mingyue Guo, Zhennan Ma, Jingni He, Ailian Wang, Xiaodan Sun, Zhen Liu

**Affiliations:** 1 Department of General Surgery, Shengjing Hospital of China Medical University, Shenyang, China.; 2 Department of Pathology, Shengjing Hospital of China Medical University, Shenyang, China.; 3 Department of Pathology, First Affiliated Hospital and College of Basic Medical Sciences, China Medical University, Shenyang, China.

**Keywords:** papillary thyroid carcinoma, Deltex3, epithelial-mesenchymal transition, AKT signal pathway, XRCC5

## Abstract

**Background:** Papillary thyroid carcinoma (PTC) is one of the most common endocrine malignant tumors. Poor prognoses such as high recurrence rate always appear in PTC patients with cervical lymph node metastasis. The process of ubiquitination plays important roles in PTC. As ubiquitin E3 ligases, Deltex (DTX) family proteins were reported to associate with multiple cancers. However, functions and mechanisms of DTX3 in PTC are currently unknown.

**Methods:** In this study, DTX3 expressions were examined in 114 PTC and paired paracancerous normal tissues through quantitative real-time polymerase chain reaction and western blot. The clinical significances of DTX3 expressions in PTC patients were also investigated. After stable transfection with either short hairpin RNA to knock down DTX3 expression or full-length complementary DNA to upregulate DTX3 expression, changes of malignant phenotypes in two PTC cell lines K1 and TPC-1 were observed using cell viability, flow cytometry, wound healing and transwell assays. Afterwards, altered expressions of epithelial-mesenchymal transition (EMT) and AKT signal pathway related proteins were measured by western blot. Immunoprecipitation and mass spectrometry (IP-MS), immunofluorescence and Co-IP were utilized to identify the possible DTX3 interacting proteins.

**Results:** Both mRNA and protein expressions of DTX3 were lower in PTC tissues and correlated with the presence of cervical lymph node metastasis (*P*<0.05). DTX3 overexpression inhibited migration and invasion of PTC cells, decreased Vimentin and phosphorylated AKT expressions, but promoted E-cadherin expression (*P*<0.05). Moreover, knockdown of DTX3 led to opposite changes (*P*<0.05). Total 46 probable DTX3 interacting proteins were identified by IP-MS. Among them, X-ray repair cross-complementing protein 5 (XRCC5) and NADH: Ubiquinone Oxidoreductase Complex Assembly Factor 5 (NDUFAF5) were verified to be associated with DTX3. Moreover, DTX3 was proved to be co-localized with XRCC5 in nucleus and promote ubiquitination of XRCC5.

**Conclusions:** DTX3 suppresses EMT by partially facilitating ubiquitination of XRCC5 to inhibit AKT signal pathway in PTC.

## Introduction

Papillary thyroid carcinoma (PTC), with increasing incidence in recent years, is the most prevalent type of thyroid carcinoma 1-3. As treatments of PTC have been greatly improved, most patients with PTC can be treated by properly applied surgery and postoperative regular thyroxine suppressive therapy alone or with postoperative radioactive iodine therapy 4-7. However, a growing body of evidence indicated that the presence of cervical lymph node metastasis varied from 69.6% to 85% in PTC patients, which was considered as an extremely high risk factor of PTC recurrence 8-10. So, it is very important to study and understand the underlying molecular mechanisms related to the metastasis of PTC.

Ubiquitination plays significant roles in tumor cells 11. Ubiquitin E3 ligase can recognize target specificity and catalyze the combination of ubiquitin with the substrate 12. Deltex (DTX) family proteins belong to ubiquitin E3 ligases 13. The classic structures of DTX family proteins are divided into three parts, including a C-terminal ubiquitin E3 ligase domain of RING finger, a motif with rich prolines and the WWE domains at N-terminus 14. Notably, the structure of DTX3 exists within a special truncated N-terminal domain lacking homology to other DTX proteins 15. DTX1, as a member of DTX proteins, has been already reported to influence the progression of tumor cells, which indicated that DTX family proteins played critical roles in tumor cells 16.

To date, little is known about the roles of DTX3 in the progression of PTC. In the present study, we evaluated DTX3 expressions in 114 PTC tissues and paired paracancerous normal tissues. Meanwhile, we compared the correlations between DTX3 expressions and clinicopathological characteristics of patients with PTC. Then, we determined the effects and mechanisms of DTX3 on cell proliferation, apoptosis, cell cycle, migration, invasion, and epithelial-mesenchymal transition (EMT) of PTC cells. In addition, we assessed the probable DTX3 interacting proteins in PTC cells. With these results, this study will enhance our understanding of DTX3 functions in PTC.

## Materials and methods

### Patients and clinicopathological data

The interactive web application of Gene Expression Profiling Interactive Analysis (GEPIA) was used to visualize the gene expressions of DTX3 in multiple human carcinomas and normal tissues of The Cancer Genome Atlas (TCGA) by box plots.

Total 114 samples of PTC patients who received surgical therapy were selected from March 2018 to March 2019 in Shengjing Hospital of China Medical University. Several exclusion and inclusion criteria were considered when selecting participants. The inclusion criteria were as follows: The age of patients was 18-80 years; Patients with PTC were all found and diagnosed for the first time; No treatment was accepted before surgery; Neck Color Doppler Ultrasound or Computed Tomography was at least evaluated before surgery; Pathological diagnosis was PTC. The exclusion criteria included cases with incomplete clinicopathological data; patients who received radiotherapy or chemotherapy before surgery; and, patients with previous thyroid surgery history.

Paracancerous tissues of the same PTC patients were collected at least 2 cm away from PTC areas. The age of patients, the tumor size and cervical lymph node metastasis were classified based on the Classification System for Differentiated Thyroid Carcinoma in the 8th edition of the American Joint Committee on Cancer (AJCC) 17. Clinical data was retrieved from patients' clinical records and presented in Table 1. All tissue specimens and clinical data were collected following protocols approved by the Ethics Committee of Shengjing Hospital of China Medical University.

### Cell culture

Human thyroid epithelial cell line Nthy-ori3-1, PTC cell lines K1 and TPC-1 were cultured. Nthy-ori3-1 and K1 were purchased from the European Collection of Cell Cultures and TPC-1 was gifted by Professor Lei Yang (China Medical University, China). K1 was cultured in a medium mixture of Dulbecco's modified Eagle's medium, Ham's F12 (Corning Life Sciences, NY, USA) and MCDB105 (Sigma-Aldrich St.Louis, Missouri) at a 1:2:1 ratio, which supplemented with 10% fetal bovine serum (FBS; Gibco Co., Grand Island, NY, USA), 100 U/mL penicillin, 100 mg/mL streptomycin, and 2 mmol/L glutamine (Sigma-Aldrich). Nthy-ori3-1 and TPC-1 were grown in Roswell Park Memorial Institute-1640 medium (Corning Life Sciences, NY, USA) and supplemented with 10% FBS, 100 U/mL penicillin, 100 mg/mL streptomycin, and 2 mmol/L glutamine in a humidified incubator with 5% CO_2_ at 37°C.

### Nuclear and cytoplasmic protein extract

Nuclear and cytoplasmic protein extracts of K1 and TPC-1 cells were separated and collected with the Nuclear and Cytoplasmic Protein Extraction Kit (P0027, Beyotime Biotechnology, China), according to the manufacturer's protocols.

### Plasmid construction and transfection

DTX3 short hairpin RNA (shRNA) and its negative control (neg-shRNA) were designed and synthesized into hU6-MCS-CMV-GFP-SV40-Neomycin plasmid (GeneChem Co., Ltd, Shanghai, China). The DTX3-shRNA target sequence is listed as 5'-GCGAGACTTCTGACATCTA-3'. The shRNA and neg-shRNA transfections were according to the manufacturer's protocols of Lipofectamine 3000 (Invitrogen, Carlsbad, CA). For stable transfection, PTC cells with G418 resistance were selected (0.5 mg/mL for K1 and 0.6 mg/mL for TPC-1).

### Lentivirus construction and transfection

Human full-length DTX3 complementary DNA (cDNA) was constructed into pSlenti-EF1a-mcherry-P2A-Puro-CMV-DTX3-3Flag lentivirus vector. PTC cells were transfected with DTX3-cDNA or neg-vector, as the negative control for DTX3-cDNA, according to the manufacturer's protocols respectively (Obio Technology Co., Ltd, Shanghai, China). For stable transfection, PTC cells with puromycin resistance were selected (2.0 mg/mL for K1 and 3.0 mg/mL for TPC-1).

### RNA extraction and quantitative real-time polymerase chain reaction (qRT-PCR)

Total RNA was extracted using Trizol (Code No. 9109, Takara Bio Inc, Japan) according to the manufacturer's instructions. cDNA was synthesized by reverse transcription using PrimeScript™ RT reagent Kit with gDNA Eraser (Code No. RR047A, Takara Bio Inc, Japan). qRT-PCR analysis was performed on a Roche LightCycler 480 II system according to the manufacturer's protocol. Primer sequences are listed in Table 2. The housekeeping gene glyceraldehyde-3-phosphate dehydrogenase (GAPDH) was used as an internal control.

### Western blot analysis (WB)

To extract total protein, RIPA Lysis Buffer (P0013B, Beyotime Biotechnology, China) with protease inhibitor phenylmethanesulfonyl fluoride (PMSF; ST506, Beyotime Biotechnology, China) and protease inhibitor cocktail (K1011, APExBIO Technology LLC) were added into tissues and cells. The homogenate was centrifuged at 14,000×g for 45 min at 4 °C. 40 μg total protein samples were separated by sodium dodecyl sulfate-polyacrylamide gel electrophoresis (SDS-PAGE) and then transferred to a polyvinylidene fluoride (PVDF) membrane. 5% bovine serum albumin (BSA) was used to block the membranes for 2 h at room temperature. Then, the membranes were incubated with specific primary antibodies overnight at 4 °C. Then, the membranes were incubated with secondary antibodies for 2 h at room temperature. Antibodies used in this study included the following: rabbit polyclonal anti-DTX3 (1:1000, cat. no. 25304-1-AP, Proteintech Group Inc), rabbit polyclonal anti-Vimentin (1:1000, cat. no. 10366-1-AP, Proteintech Group Inc), mouse monoclonal anti-E-cadherin (1:2000, cat. no. 60335-1-Ig, Proteintech Group Inc), rabbit polyclonal anti-N-cadherin (1:2000, cat. no. 22018-1-AP, Proteintech Group Inc), rabbit monoclonal anti-AKT (1:1000, cat. no. #4691, Cell Signaling Technology Inc), rabbit monoclonal anti-Phospho-AKT (1:2000, cat. no. #4060, Cell Signaling Technology Inc), rabbit monoclonal anti-NDUFAF5 (1:1000, cat. no. ab192235, Abcam Inc), rabbit polyclonal anti-GAPDH (1:10000, cat. no. 10494-1-AP, Proteintech Group Inc), rabbit monoclonal anti-Histone H3 (1:2000, cat. no. #4499, Cell Signaling Technology Inc), rabbit polyclonal anti-tubulin (1:10000, cat. no. 11224-1-AP, Proteintech Group Inc), rabbit polyclonal anti-XRCC5 (1:1000, cat. no. 16389-1-AP, Proteintech Group Inc), rabbit polyclonal anti-ubiquitin (1:1000, cat. no. 10201-2-AP, Proteintech Group Inc) and peroxidase-conjugated goat anti-rabbit or anti-mouse IgG (H+L) (1:3000, Zhongshan Goldenbridge Company, Beijing, China) as the secondary antibody. Enhanced chemiluminescence kit (P0018AS, Beyotime Biotechnology, China) was performed to show the binding conditions between antigens and antibodies. The integrated optical density (IOD) of each band was measured with the Image-Pro Plus 6.0 software (Media Cybernetics Inc., Rockville, MD). The ratio between the IOD of target protein and internal control in the same sample was calculated as the relative protein expression of the target protein.

### Cell viability assay

96-well plates were seeded in 3×10^3^ cells per well. 20 μL 3-(4,5-dimethylthiazol-2-yl)-5-(3-carboxymethoxyphenyl)-2-(4-sulfophenyl)-2H-tetrazolium (MTS; Promega, USA) was added into designated wells and incubated at 37 °C for 4 h in a humidified, 5% CO_2_ atmosphere. After 10 s of vortexing, the optical density (OD) value at a wavelength of 490 nm was measured and indicated the viability of cells. The average OD value represented five individual wells.

### Flow cytometry analysis

Cells were harvested and washed with phosphate-buffered saline (PBS) for twice. For cell apoptosis, Annexin V-PE and 7-aminoactinomycin D (7-AAD) solution (Keygentec, Nanjing, China) were added to each sample for incubating 15 min in the dark. For cell cycle analysis, cells were mixed with 70% ethanol for overnight at 4 °C, washed with cold PBS and centrifugally harvested at 1000 rpm for 5 min. The supernatants were resuspended in Propidium Iodide (PI) and Ribonuclease A (RNase A) staining buffers (Keygentec, Nanjing, China) for 15-20 min in the dark. The fluorescent signals were detected by flow cytometry (BD FACSCalibur) within 1 h. Living cells were in PE-/7-AAD- quadrant, early apoptotic cells were in PE+/7-AAD- quadrant and late apoptotic cells were in PE+/7-AAD+ quadrant.

### Wound-healing assay

When cells were cultured 95-100% confluence in 6-well plates, 200 μL pipette tips were selected to scratch the cell monolayer to make a straight gap. After washing twice with PBS, cells remained in well were incubated in serum-free medium to diminish cell proliferation for 24 h. At both 0 h and 24 h, the wounded gaps were photographed with a light microscope (Nikon E100, Japan), at 100× magnification. The average cell migration areas were measured and analyzed from 5 different areas for each wound. The percentage of wound closure was calculated as followed: (original denuded area at 0 h - actual denuded area at 24 h) / original denuded area at 0 h×100%.

### Matrigel invasion assay

24-well Transwell chambers with an 8 mm pore size (Corning Incorporated, NY, USA) were precoated with Matrigel (BD Biosciences, Bedford, MA). 600 μL medium supplemented with 10% FBS was added to each lower chamber. 3×10^4^ cells in 200 μL serum-free medium were seeded in each upper chamber and cultured for 24 h. Fixed with 4% paraformaldehyde for 15 min and stained with 0.1% crystal violet for 15 min, cells invaded to the lower surface of the membrane were pictured and counted in 5 random fields (200× magnification).

### Immunoprecipitation and mass spectrometry (IP-MS)

Cells were harvested and lysed in IP lysis buffer (cat. no. P0013, Beyotime Biotechnology, China) with PMSF and protease inhibitor cocktail for 30 min at 4 °C. Samples were then centrifuged at 14,000×g for 45 min at 4 °C. 20 μL of supernatant was saved as the “input”. Either primary antibody or normal IgG antibody was added to the remaining cell lysis and then gently rotated overnight at 4 °C. Primary antibodies included: mouse monoclonal anti-DTX3 antibody (cat. no. sc-376439, Santa Cruz Biotechnology, Inc), rabbit polyclonal anti-XRCC5 (cat. no. 16389-1-AP, Proteintech Group Inc), rabbit monoclonal anti-NDUFAF5 (cat. no. ab192235, Abcam Inc), normal mouse IgG antibody (cat. no. A7028, Beyotime Biotechnology, China) and normal rabbit IgG antibody (cat. no. #2729, Cell Signaling Technology Inc). 20 μL Protein A/G PLUS-Agarose beads (cat. no. sc-2003, Santa Cruz Biotechnology, Inc) were added to the cell lysate and then rotated for 2 h at 4 °C. The beads were washed with IP lysis buffer and centrifuged at 3000 rpm for 5 min to harvest. After washed 4 times, the beads were boiled with either 120 μL SDT buffer (4% SDS, 100 mM Tris-HCl, 1 mM DTT, pH 7.6) or 20 μL 2×loading buffer for 10 min at 100 °C. Tubes were centrifuged at 14,000×g for 10 min and the supernatants were transferred to new tubes at 4 °C. The solution in SDT buffer was prepared for MS analysis (Shanghai Applied Protein Technology Co., LTD). The solution in 2×loading buffer was prepared for WB.

### Immunofluorescence

4×10^4^ cells per well were cultured in 24-well plates. After adherence, cells were fixed by 4% paraformaldehyde for 15 min at room temperature. After washed with PBS, cells were blocked in 5% normal goat serum with 0.3% Triton-X100 for 30 min. Cells were incubated with a mixture of primary antibodies for overnight at 4℃ after washed with PBS. The primary antibodies used were as follows: mouse monoclonal anti-DTX3 (1:100, cat. no. sc-376439, Santa Cruz Biotechnology, Inc) and rabbit polyclonal anti-XRCC5 (1:200, cat. no. 16389-1-AP, Proteintech Group Inc). Then cells were washed with PBS 3 times for 5 min and incubated with a mixture of secondary antibodies, goat anti-mouse IgG Dylight 594 (1:100) and goat anti-rabbit IgG Dylight 488 (1:100), for 1 h. For nuclei staining, cells were counterstained with DAPI (cat. no. C0065, Solarbio Life Sciences) for 5 min and mounted with Antifading Mounting Medium (cat. no. S2100, Solarbio Life Sciences) for confocal image acquisition (400× magnification).

### Bioinformation analysis

WEB-based GEne SeT AnaLysis Toolkit (WebGestalt) were utilized to visualize Gene ontology (GO) and the Kyoto Encyclopedia of Genes and Genomes (KEGG) enrichment analysis 18. The Search Tool for the Retrieval of Interacting Genes (STRING) database was visualized for protein-protein interaction (PPI). The cBioPortal for Cancer Genomics was analyzed the co-expression genes of DTX3 in PTC 19-21.

### Statistical analysis

Each experiment was performed at least three times. Data is provided as mean ± standard deviation. SPSS 24.0 statistical software for Windows (SPSS, Inc., Chicago, IL, USA) was used for analysis. Graphing was performed in GraphPad Prism 7. The T-test was used to assess the results of qRT-PCR and WB expressions of DTX3 in PTC and paired paracancerous normal tissues and applied to calculate correlations between DTX3 expressions and clinicopathologic characteristics of PTC. Multiple group comparison's test, such as ANOVA, was used to compare the differences. When *P*<0.05, the results were considered statistically significant.

## Results

### The expressions of DTX3 are lower in multiple carcinomas including PTC and closely associated with cervical lymph node metastasis of patients with PTC

**Figure 1:**
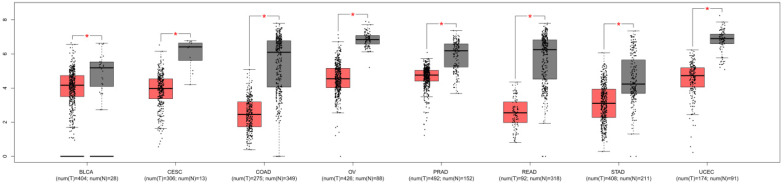
**The expressions of DTX3 in multiple carcinomas of TCGA database.** T: tumor; N: normal tissue; BLCA: bladder urothelial carcinoma; CESC: cervical squamous cell carcinoma and endocervical adenocarcinoma; COAD: colon adenocarcinoma; OV: ovarian serous cystadenocarcinoma; PRAD: prostate adenocarcinoma; READ: rectum adenocarcinoma; STAD: stomach adenocarcinoma; UCEC: uterine corpus endometrial carcinoma; UCS: uterine carcinosarcoma. *P < 0.05.

**Figure 2:**
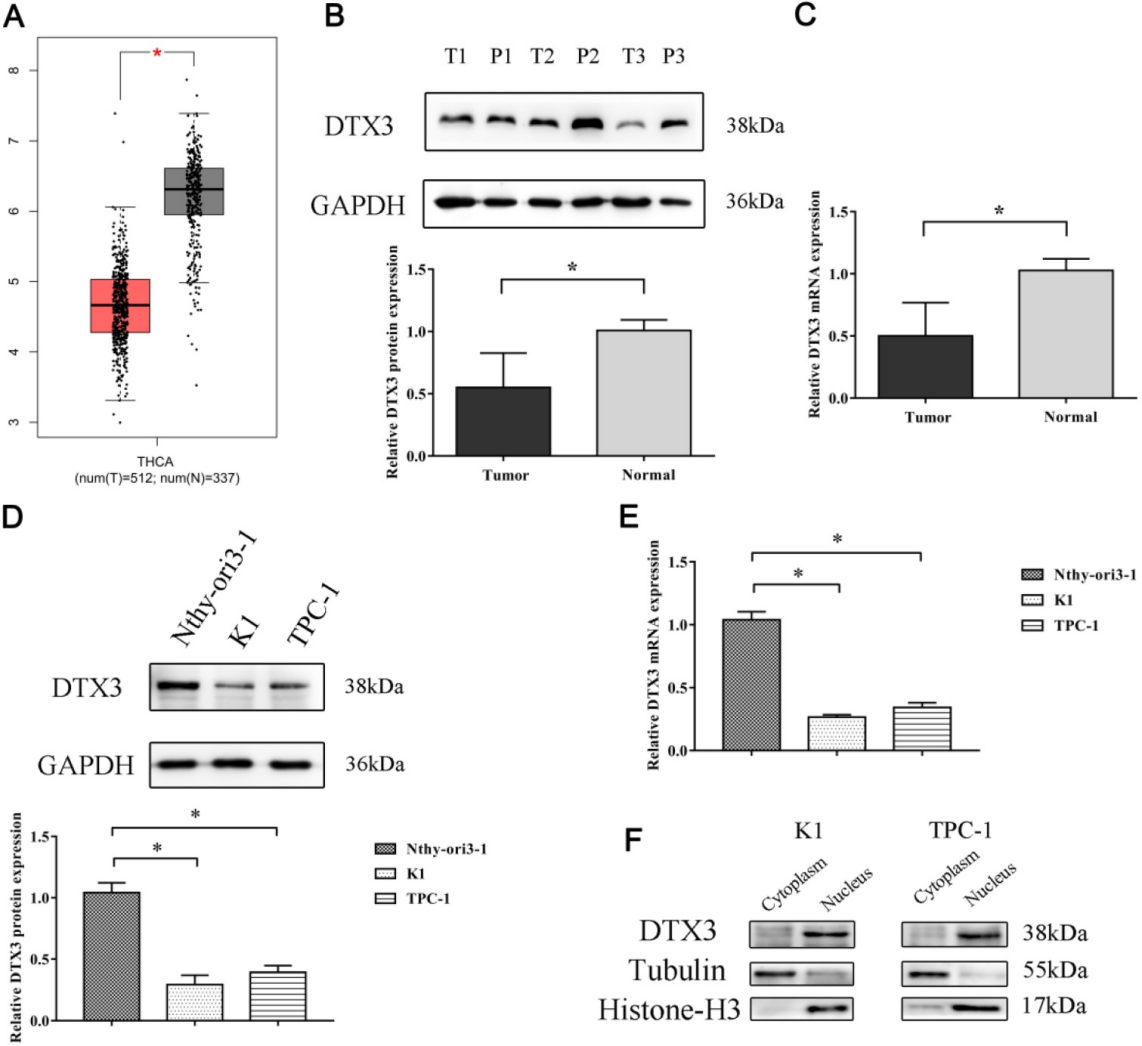
** mRNA and protein relative expressions of DTX3 in PTC tissues, PTC cell lines and peritumoral nonmalignant tissues and cell line.** (A) DTX3 gene expressions in thyroid carcinoma (T) and normal tissues (N) of TCGA database. (B) Relative protein expressions of DTX3 in PTC tissues and peritumoral nonmalignant tissues. Lower panel: Integrated optical density (IOD) of DTX3 protein was presented as relative expression level after normalization with internal control GAPDH. (C) Relative mRNA expressions of DTX3 in PTC tissues and peritumoral nonmalignant tissues. (D) Relative protein expressions of DTX3 in human thyroid epithelial cell line Nthy-ori3-1, PTC cell lines K1 and TPC-1. Lower panel: IOD of DTX3 protein was presented as relative expression level after normalization with internal control GAPDH. (E) Relative mRNA expressions of DTX3 in human thyroid epithelial cell line Nthy-ori3-1, PTC cell lines K1 and TPC-1. (F) The subcellular localization of DTX3 in K1 and TPC-1 cells. Histone H3 was used as internal control of nucleus and tubulin was used as internal control of cytoplasm. *P < 0.05.

To explore the significances of DTX3 in PTC, we searched GEPIA websites to compare the expressions of DTX3 in different carcinomas and normal tissues of TCGA database. As shown in Figure 1, the results of box plots showed that the expressions of DTX3 were significantly lower in at least 8 different kinds of carcinomas than those in paired normal tissues, such as bladder urothelial carcinoma (BLCA), colon adenocarcinoma (COAD), ovarian serous cystadenocarcinoma (OV) and prostate adenocarcinoma (PRAD) (*P*<0.05). The result of box plot for thyroid carcinoma was also similar and shown in Figure 2A.

According to the exclusion and inclusion criteria, 114 PTC and paired paracancerous normal tissues were collected in Shengjing Hospital of China Medical University to detect both mRNA and protein relative expressions of DTX3. The results of qRT-PCR and WB proved that the relative mRNA and protein expressions of DTX3 in PTC tissues were 0.501 ± 0.267 and 0.551 ± 0.277, which were markedly lower as compared to those in the paired paracancerous normal tissues (1.026 ± 0.094 and 1.008 ± 0.084, *P*<0.05) (Figures 2B and 2C).

Then, we analyzed the correlations between clinicopathological characteristics and DTX3 expressions in PTC. As showed in Table 1, relative mRNA and protein expressions of DTX3 in group with cervical lymph node metastasis were 0.444 ± 0.254 and 0.490 ± 0.255, which were prominently lower than those in no cervical lymph node metastasis group (0.607 ± 0.261 and 0.663 ± 0.282, *P*<0.05). Other clinicopathological characteristics, such as age and sex of patients, multifocality and extra-capsular invasion of PTC, were not associated with the relative expressions of DTX3 (*P*>0.05). These findings suggested that the low expression of DTX3 might play important roles in the metastasis of PTC.

### The expressions of DTX3 are lower in PTC cell lines and mainly located in nucleus

To explore the exact roles of DTX3 in PTC cells, we compared the relative mRNA and protein expressions of DTX3 in two PTC cell lines (K1 and TPC-1) and thyroid epithelial cell line Nthy-ori3-1 by qRT-PCR and WB. The results showed that the relative mRNA and protein expressions of DTX3 in K1 and TPC-1 were both significantly lower than those in Nthy-ori3-1 (*P*<0.05) (Figures 2D and 2E).

To detect the subcellular locations of DTX3 in PTC cell lines, the nuclear and cytoplasmic protein extracts of both K1 and TPC-1 cells were prepared for WB analysis. The outcomes indicated that the majority of DTX3 was distributed in the nucleus (Figure 2F).

### DTX3 suppresses migration and invasion of PTC cells

**Figure 3:**
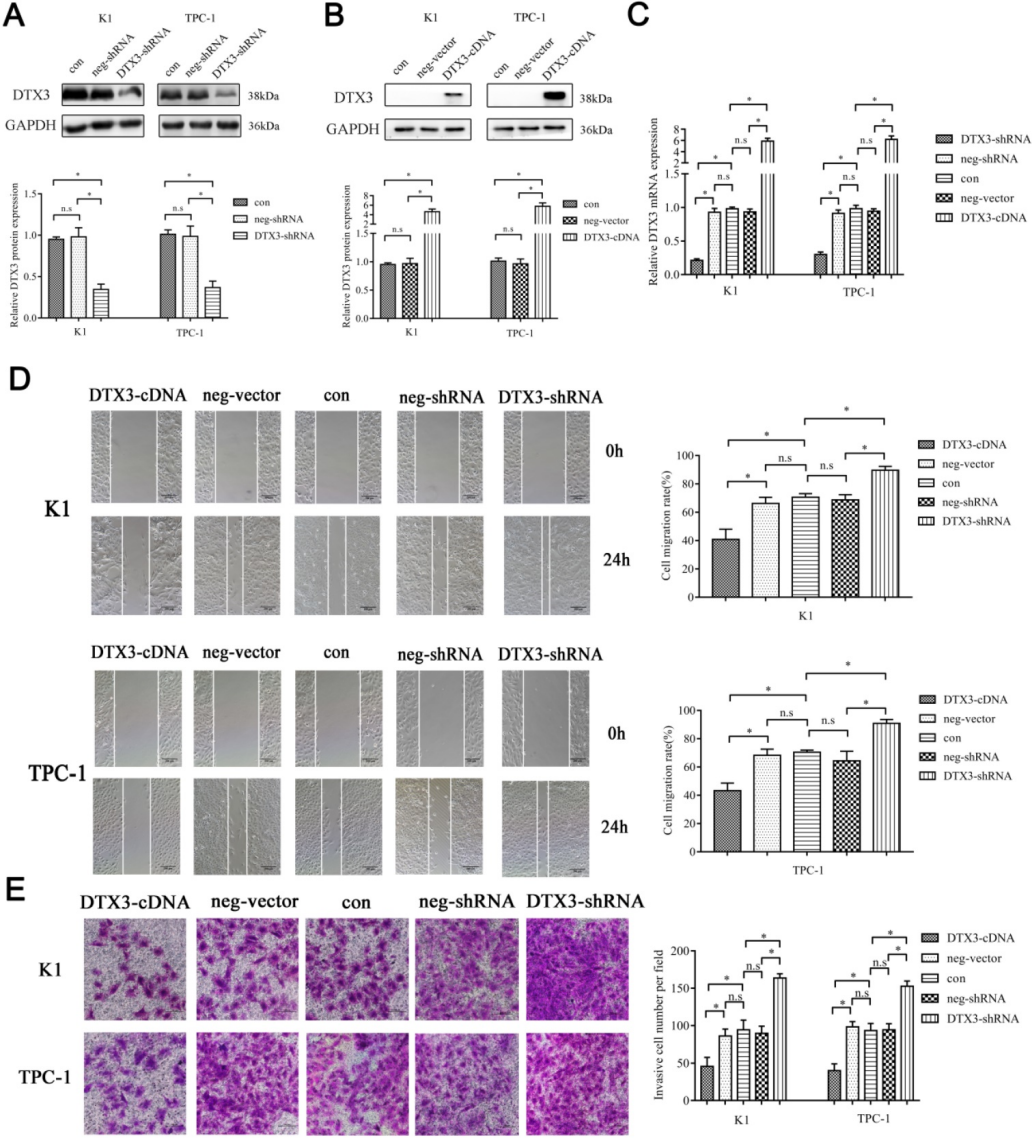
**The influences of upregulation and downregulation of DTX3 expressions on migration and invasion of PTC cells.** (A) DTX3 relative downregulated protein expression levels were measured by western blot using anti-DTX3 antibody. GAPDH was used as a loading control. Lower panel: Integrated optical density (IOD) of DTX3 protein was presented as relative expression level after normalization with internal control. (B) DTX3 relative upregulated protein expression levels were measured by western blot using anti-DTX3 antibody. GAPDH was used as a loading control. Lower panel: IOD of DTX3 protein was presented as relative expression level after normalization with internal control. (C) DTX3 relative upregulated and downregulated mRNA expression levels were measured by qRT-PCR. (D) The migration abilities affected by upregulation and downregulation of DTX3 were measured by wound healing assay (100× magnification). (E) The invasion abilities affected by upregulation and downregulation of DTX3 were measured by Matrigel-coated transwell invasion assay (200× magnification). *P < 0.05, n.s means not significant.

We transfected K1 and TPC-1 cells with DTX3-shRNA, DTX3-cDNA, neg-shRNA, or neg-vector respectively. K1 and TPC-1 cells with DTX3 overexpression and downregulation were stably transfected and confirmed by qRT-PCR and WB. In K1 DTX3-shRNA group, the relative mRNA and protein expressions of DTX3 were 0.213 ± 0.023 and 0.346 ± 0.064, which were both significantly reduced, compared with control groups (0.928 ± 0.057 and 0.981 ± 0.109, *P*<0.05). In TPC-1 DTX3-shRNA cells, the relative mRNA and protein expressions of DTX3 were 0.301 ± 0.035 and 0.368 ± 0.078, which were both obviously lower than those in control groups (0.911 ± 0.050 and 0.985 ± 0.126, *P*<0.05). At the same time, In K1 DTX3-cDNA group, the relative mRNA and protein expressions of DTX3 were 5.867 ± 0.532 and 4.612 ± 0.570, which were both upregulated in comparison with control groups (0.933 ± 0.044 and 0.967 ± 0.094, *P*<0.05). In TPC-1 DTX3-cDNA cells, the relative mRNA and protein expressions of DTX3 were 6.169 ± 0.637 and 5.761 ± 0.719, which were both overexpressed than those in control groups (0.943 ± 0.037 and 0.963 ± 0.085, *P*<0.05) (Figures 3A, 3B and 3C).

Then, we performed MTS assay, flow cytometry, wound-healing assay and Matrigel invasion experiments to assess the effects of DTX3 on malignant phenotypes of PTC cells. In the wound-healing assay, the percentages of wound closure in K1 and TPC-1 DTX3-cDNA cells were 40.899% ± 7.110% and 43.261% ± 5.328%, which were smaller than those in control groups (66.235% ± 4.277% and 68.313% ± 4.303%, *P*<0.05). While, the percentages of wound closure in K1 and TPC-1 DTX3-shRNA cells were 89.648% ± 2.751% and 90.867% ± 2.720%, which were larger as compared with control groups (68.701% ± 3.696% and 64.409% ± 6.730%, *P*<0.05) (Figure 3D).

In the Matrigel invasion experiments, the average numbers of cells passing through in K1 and TPC-1 DTX3-cDNA groups were 45.667 ± 12.014 per field and 41.333 ± 7.506 per field, which were less than those in control groups (86.333 ± 9.074 per field and 98.333 ± 7.024 per field, *P*<0.05). However, the average invaded cells in K1 and TPC-1 DTX3-shRNA groups were 163.667 ± 5.686 per field and 152.667 ± 7.024 per field, which were larger than those in control groups (89.667 ± 9.504 per field and 94.333 ± 8.327 per field, *P*<0.05) (Figure 3E). The changes in cell viability, cell apoptosis and cell cycle assays among DTX3-cDNA, DTX3-shRNA and control groups were not significantly different (*P*>0.05, data not shown).

These results suggested that DTX3 overexpression reduced cell migration and invasion in PTC cells, and inhibition of DTX3 expression led to reverse changes.

### DTX3 inhibits EMT in PTC cells

**Figure 4:**
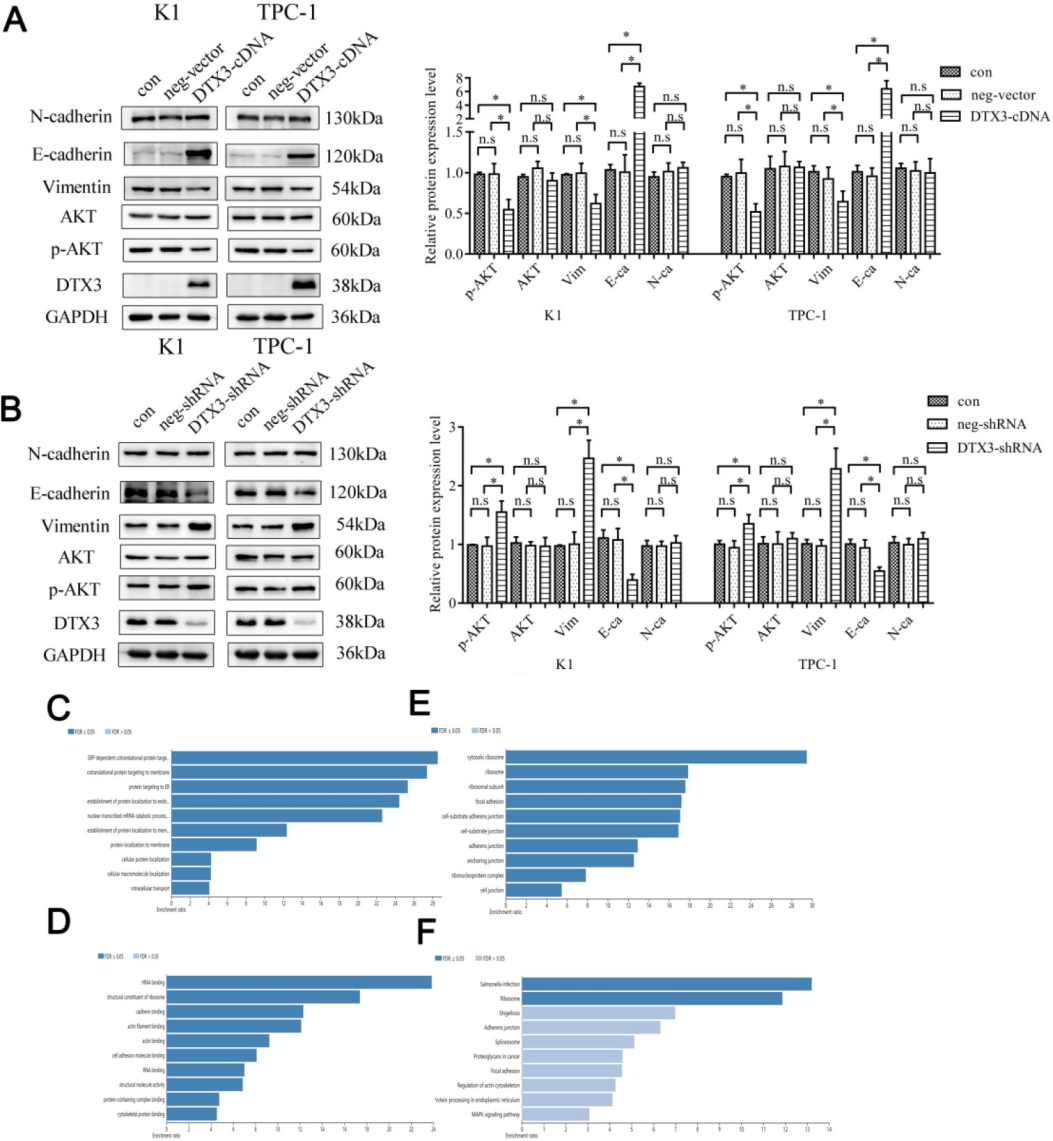
**DTX3 inhibits EMT partially by regulating AKT signal pathway in PTC cells and each top 10 GO and KEGG enrichment analysis terms of 46 DTX3 interacting proteins.** (A) Comparisons of expressions of EMT and AKT signal pathway related proteins in K1 and TPC-1 cells transfected with DTX3 cDNA by western blot. Right panel: Relative expression levels indicated are normalized to GAPDH levels. (B) Comparisons of expressions of EMT and AKT signal pathway related proteins in K1 and TPC-1 cells transfected with DTX3 shRNA by western blot. Right panel: Relative expression levels indicated are normalized to GAPDH levels. (C) Top 10 biological process of GO enrichment terms. (D) Top 10 molecular function of GO enrichment terms. (E) Top 10 cellular components of GO enrichment terms. (F) Top 10 KEGG pathway enrichment terms. GAPDH protein was used as a loading control. *P < 0.05, n.s means not significant.

The relative protein expressions of Vimentin, E-cadherin and N-cadherin were evaluated by WB. In K1 and TPC-1 DTX3-cDNA cells, the relative protein expressions of Vimentin were 0.620 ± 0.113 and 0.643 ± 0.129, which were lower than those in control groups (0.992 ± 0.122 and 0.989 ± 0.088, *P*<0.05). In K1 and TPC-1 DTX3-cDNA cells, the relative E-cadherin expressions were 6.736 ± 0.508 and 6.384 ± 1.218, which were higher than those in control groups (1.006 ± 0.214 and 0.955 ± 0.101, *P*<0.05). In K1 and TPC-1 DTX3-shRNA groups, the relative protein expressions of Vimentin and were 2.466 ± 0.309 and 2.287 ± 0.351, which were higher than those in control groups (1.004 ± 0.207 and 0.973 ± 0.105, *P*<0.05). In K1 and TPC-1 DTX3-shRNA groups, E-cadherin relative expressions were 0.394 ± 0.096 and 0.547 ± 0.066, which were much lower than those in control groups (1.077 ± 0.195 and 0.942 ± 0.134, *P*<0.05). However, the change of N-cadherin by DTX3 was not obviously observed in K1 and TPC-1 cells (*P*>0.05) (Figures 4A and 4B).

Above all, DTX3 acted pivotal parts in suppressing the migration and invasion of PTC cells by downregulation of EMT.

### The inhibitory effects of DTX3 on EMT are partially regulated by AKT signal pathway

Since AKT signal pathway was closely associated with EMT, we wondered if some proteins in AKT signal pathway would be influenced by DTX3. AKT and phosphorylated AKT (p-AKT) expressions were evaluated by WB. In K1 and TPC-1 DTX3-cDNA cells, the relative protein expressions of p-AKT were 0.549 ± 0.124 and 0.518 ± 0.099, which were lower than those in control groups (0.982 ± 0.129 and 0.995 ± 0.168, *P*<0.05). In K1 and TPC-1 DTX3-shRNA groups, the relative protein expressions of p-AKT were 1.552 ± 0.186 and 1.350 ± 0.157, which were higher than those in control groups (0.970 ± 0.151 and 0.943 ± 0.117, *P*<0.05). The changes of AKT by DTX3 was not obviously observed in K1 and TPC-1 cells (*P*>0.05) (Figures 4A and 4B).

These results suggested that DTX3 in PTC cells inhibited the EMT process partially through AKT signal pathway.

### Identification and functional enrichments of DTX3 interacting candidates

As an ubiquitin E3 ligase, IP-MS was performed to recognize the interacting proteins with DTX3. According to the results, there were 11 unique peptides corresponding to DTX3 protein, which suggested that DTX3 was successfully immunoprecipitated and captured by MS. Then, to reduce the influence from potential contamination and/or nonspecific binding of the IgG portion of DTX3 antibody as much as possible, the candidates for DTX3 interacting proteins were screened as following criteria: proteins with at least 2 unique peptides; proteins were uniquely identified in DTX3-IP group; proteins in DTX3-IP group with unique peptides number 2 times more than those of IgG-IP negative control group 22. After selection, a total of 46 proteins were identified as putatively interacting with DTX3 (Table 3).

To figure out the molecular functions and mechanisms of these DTX3 interacting proteins, the enrichments of GO and KEGG pathway were carried on. Each top 10 GO and KEGG enrichment analysis terms were listed. According to the GO enrichment results, these proteins based on biological process were mainly classified in SRP-dependent cotranslational protein targeting to membrane (GO: 0006614) (Figure 4C). In molecular function enrichment, these proteins were mostly enriched in rRNA binding (GO: 0019843) (Figure 4D). The enriched proteins were in some cellular components including cytosolic ribosome (GO: 0022626) (Figure 4E). KEGG pathway enrichment implied that proteins interacting with DTX3 were mainly enriched in pathways such as ribosome (hsa03010) (Figure 4F).

### DTX3 is co-localized with XRCC5 and promotes its ubiquitination

**Figure 5:**
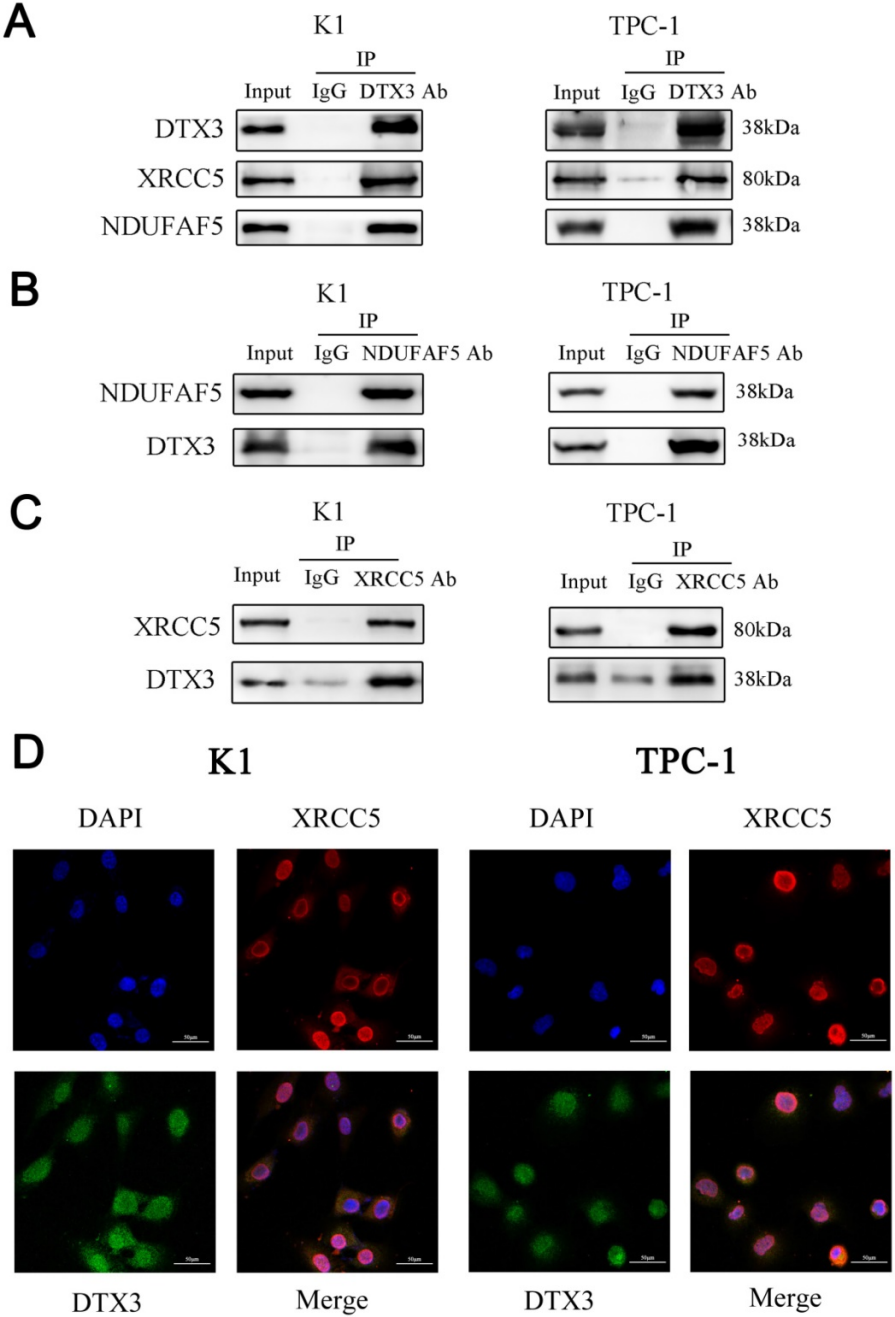
**NDUFAF5 and XRCC5 were interacted with DTX3 respectively and XRCC5 was co-localization with DTX3.** (A) Whole cell lysates were prepared and subjected to immunoprecipitation with either DTX3 antibody or normal IgG antibody. Antibodies of XRCC5 and NDUFAF5, as primary antibodies, were used to western blot. (B) Whole cell lysates were prepared and subjected to immunoprecipitation with either NDUFAF5 antibody or normal IgG antibody. DTX3 antibody as the primary antibody was used to western blot. (C) Whole cell lysates were prepared and subjected to immunoprecipitation with either XRCC5 antibody or normal IgG antibody. DTX3 antibody as the primary antibody was used to western blot. (D) Dual immunofluorescence staining for the co-localization of DTX3 and XRCC5 in PTC cells (400× magnification).

To identify IP-MS results of DTX3 interacting proteins much more accurate, we searched protein-protein interaction (PPI) of DTX3 using the STRING database and co-expression genes of DTX3 in the cBioPortal. As combined PPI and co-expression genes of DTX3 with IP-MS results, the intersection called our attentions to two proteins named XRCC5 (X-ray repair cross-complementing protein 5) and NDUFAF5 (NADH: Ubiquinone Oxidoreductase Complex Assembly Factor 5). Next, Co-IP analysis showed that endogenous DTX3 could interact with either NDUFAF5 or XRCC5 (Figures 5A, 5B and 5C).

To see the co-localizations of DTX3 and XRCC5, immunofluorescence was performed. The results showed both DTX3 (Green) and XRCC5 (Red) were found to be present as mainly nuclear localizations. Moreover, they were co-localized since a clear yellow overlap was depicted (Figure 5D). These demonstrated the co-localization of DTX3 and XRCC5 in PTC cells.

**Figure 6:**
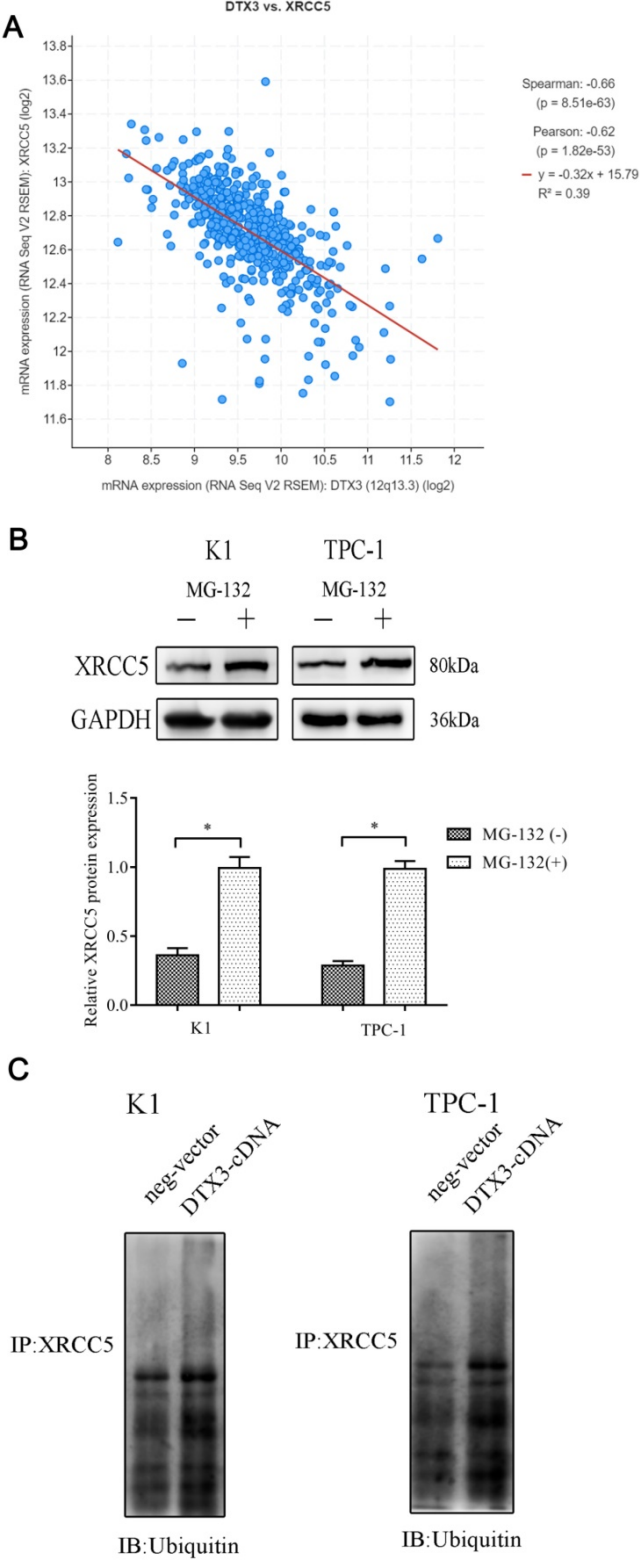
**The degradation of XRCC5 was ubiquitin/proteasome-dependent and DTX3 could promote the ubiquitination of XRCC5.** (A) The negative co-expression relationship between DTX3 and XRCC5 in PTC database of TCGA. (B) Cells were treated with 10 µM MG-132 for 6 h and whole cell lysates were prepared to western blot with XRCC5 antibody. Lower panel: Integrated optical density (IOD) of XRCC5 protein was presented as relative expression level after normalization with internal control GAPDH. (C) Cells were treated with 10 µM MG-132 for 6 h and whole cell lysates were subjected to immunoprecipitation with either XRCC5 antibody or normal IgG antibody. Ubiquitin antibody was used as the primary antibody to western blot.

According to the co-expression result from the cBioPortal, we figured out that there was a negative co-expression correlation between DTX3 and XRCC5 (Figure 6A). We supposed that DTX3 might be involved in the protein degradation of XRCC5. To confirm this speculation, proteasome inhibitor MG-132 was used to suppress proteasome functions in PTC cells. With MG-132, the relative protein expressions of XRCC5 were higher than those in cells without MG-132 added (*P*<0.05) (Figure 6B). Then, to evaluate the effects of DTX3 on the proteasome dependent degradation of XRCC5, MG-132 was used and the expressions of ubiquitinated XRCC5 were detected. In K1 and TPC-1 DTX3-cDNA cells, the relative protein expressions of ubiquitinated XRCC5 were higher than those in the control groups (*P*<0.05) (Figure 6C).

Altogether, these results indicated that DTX3 could interact with XRCC5 and upregulate its ubiquitination.

## Discussion

Ubiquitin E3 ligases play key roles in the ubiquitination pathway of cells. As one kind of ubiquitin E3 ligases, four members of DTX family proteins (DTX1, DTX2, DTX3 and DTX4) have been identified so far in human beings. Recently, more evidence discovered the functions of DTX family proteins in several tumors. The migration and invasion of Glioblastoma (GBM) cells were positively associated with expressions of DTX1. Patients of GBM with a lower level of DTX1 survived longer and had better prognoses 23. In diffuse large B-cell lymphoma (DLBCL), mutations in DTX1 gene could be discovered to impair its original effects, nearly 65% of which were found in the WWE1-domain 24. In gastric cancer, DTX1 was reported specifically down-regulated and promoted cellular FLICE inhibitory protein (c-FLIP), as its E3 ligase substrate, to degrade through the endosome-lysosomal pathway 25. DTX4 was significantly up-regulated through comparison of the interaction network between miRNAs and target genes in nasopharyngeal carcinoma samples 26.

In humans, the gene of DTX3 is in chromosome 12 (12q13.3). The protein of DTX3 consists of 347 amino acids and the molecular weight is 37988 Da. DTX3 has received much attention in the past decade. It was discussed that the transcriptional change of DTX3 was a direct consequence of the activation of E2F transcription factors and regulated the expansion of hepatocellular carcinoma cells 27. There was a significant relationship between DNA copy number alteration and mRNA expression of DTX3 in luminal subtype breast cancer. Gene amplification of DTX3 was also proved to have impacts on overall survival of luminal subtype breast cancer 28. It was reported that the expression of DTX3, acted as an anti-oncogene, was negatively correlated with NOTCH2 and suppressed proliferation and tumorigenicity of human esophageal carcinoma cells 29.

However, the impacts of DTX3 in the progression of PTC are still unknown. In present study, we showed DTX3 was much lower in PTC than in paired normal tissues in both mRNA and protein expressions (*P*<0.05). Moreover, there were prominently correlations between DTX3 expressions and the presence of cervical lymph node metastasis in PTC (*P*<0.05). After manipulating DTX3 expressions in PTC cells, we proved that downregulation of DTX3 facilitated the migration and invasion of PTC cells, while upregulation of DTX3 repressed those behaviors. These results suggested that DTX3 was an anti-oncogene and suppressed the migration and invasion of PTC.

It is generally considered that an important mechanism of migration and invasion of cancer cells lies in EMT 30. The process of EMT includes the epithelial cell depolarization and loss of adhesion, cytoskeleton transforming and alteration to morphological characteristics of mesenchymal cells 31. When EMT occurred, E-cadherin was downregulated with upregulated Vimentin and N-cadherin 32. In our study, the diminution of DTX3 led to markedly decline of E-cadherin expression, while obviously elaborated Vimentin expression. And DTX3 overexpression caused reverse phenomena. These results showed that DTX3 attenuated the process of EMT to abate the migration and invasion of PTC cells.

A close relationship between EMT and AKT signal pathway has been reported in the literatures 33. In our study, the increased phosphorylated AKT was observed after DTX3 expression was knocked down. When DTX3 was overexpressed, the expression of phosphorylated AKT was downregulated. These results demonstrated that DTX3 suppressed AKT signal pathway which participating the restrain of EMT process.

Two or more proteins form complexes through noncovalent bonds to constitute cell biochemical reaction network. Exploring and understanding the association between two interacting proteins is of great significance to analyze the regulation mechanisms in tumor cells. In this study, 46 interacting partners of DTX3 were identified and chosen by IP-MS. The functional enrichment results suggested that these DTX3 interacting proteins were associated with biological processes of transportation and localization. Binding with RNA, actin and cytoskeleton protein might be the main molecular functions of these DTX3 interacting proteins.

As the negative co-expression between DTX3 and XRCC5 in PTC database of TGCA was noticed, we focused on exploring the mechanism of XRCC5 regulated by DTX3. XRCC5, also called Ku80, forms a heterodimer with XRCC6 to bind the ends of broken DNA double strands. The heterodimer is also considered as participating in migration and invasion of tumors, not just maintaining the stability of chromosomes 34. Recently, XRCC5 has been reported to be highly expressed in various tumors such as gastric cancer, breast cancer and hepatocellular carcinoma 35-37. In PTC cells, XRCC5 was also showed high expression. Knocking down XRCC5 decreased the malignancy and inactivated the AKT signal pathway in PTC cells 38. However, it has not yet been established whether XRCC5 can be regulated by DTX3. In this paper, we shed new light on the interaction and co-localizations of DTX3 and XRCC5. We also took a new look at the mechanism that DTX3 inhibited EMT of PTC cells by partially promoting ubiquitination of XRCC5 to restrain the AKT signal pathway.

Compared with PPI results of DTX3 from STRING, NDUFAF5 was considered as another DTX3 interacting protein of high reliability 39. NDUFAF5, a member of seven-β-strand S-adenosylmethionine-dependent methyltransferases, which is totally located in the mitochondria of mammal cells. Complex I (NADH ubiquinone oxidoreductase) in mitochondrion is assembled from forty-four different proteins with the main functions of transferring the electrons of NADH along the respiratory chain and storing the energy in the form of ATP. NDUFAF5 was reported to catalyze the hydroxylation of Arg-73 in NADH: Ubiquinone Oxidoreductase Core Subunit S7 (NDUFS7) and affect the biogenesis of Complex I at an early stage of assembly 40. Mutations in NDUFAF5 were found to be related to cavitating leukoencephalopathies, lactic acidosis, classic Leigh syndrome, hyponatremia and some lethal neonatal mitochondrial diseases 41-43. In our study, Co-IP assay was performed and the results proved that there was an endogenous interaction between DTX3 and NDUFAF5 in PTC cells.

## Conclusions

In the present study, we showed DTX3 expressed low in PTC tissues both in mRNA and protein significantly. DTX3 expressions were relevant to the cervical lymph node metastasis of patients with PTC. DTX3 exerted the anti-oncogenic effects on EMT in PTC cells, which were partially mediated by interacting with XRCC5 to promote its ubiquitination to regulate AKT signal pathway. However, more exact molecular mechanisms of DTX3 interacting XRCC5 requires further study.

## Figures and Tables

**Table 1:** The correlation analysis of DTX3 mRNA and protein expressions in patients with PTC

Parameters	Total	mRNA	*P*-value	Protein	*P*-value
**Age (years)**			0.610		0.415
<55	76	0.510±0.272		0.566±0.267	
≥55	38	0.483±0.259		0.521±0.296	
**Gender**			0.438		0.498
Male	32	0.532±0.280		0.579±0.294	
Female	82	0.489±0.262		0.540±0.271	
**Tumor diameter**			0.299		0.138
≤ 2 cm	48	0.532±0.245		0.596±0.263	
> 2 cm	66	0.479±0.281		0.518±0.284	
**Cervical lymph node metastasis**			0.002*		0.001*
No	40	0.607±0.261		0.663±0.282	
Yes	74	0.444±0.254		0.490±0.255	
**Multifocality**			0.919		0.947
No	61	0.499±0.270		0.552±0.274	
Yes	53	0.504±0.265		0.549±0.282	
**Extra-capsular invasion**			0.137		0.281
No	69	0.531±0.281		0.573±0.280	
Yes	45	0.455±0.239		0.516±0.271	

*P<0.05.

**Table 2:** Primers used in this study

Gene	Primer sequence	Product size (bp)
DTX3	Forward: 5'- CCAGCGTCTCACCTTCACTATCG -3'	86
	Reverse: 5'- TGGTCTTGTGGTGGATGTCGTTC -3'	
GADPH	Forward: 5'-GCACCGTCAAGGCTGAGAAC-3'	138
	Reverse: 5'-TGGTGAAGACGCCAGTGGA-3'	

**Table 3:** Details of 46 candidates for interacting with DTX3

Accession	Gene name	Description
Q60FE6	FLNA	Filamin A
O95425	SVIL	Supervillin
Q01082	SPTBN1	Spectrin beta chain, non-erythrocytic 1
Q00610	CLTC	Clathrin heavy chain 1
O75369	FLNB	Filamin-B
O15144	ARPC2	Actin-related protein 2/3 complex subunit 2
B4E0E1	PARP1	Poly [ADP-ribose] polymerase 1
Q8NEN9	PDZD8	PDZ domain-containing protein 8
E9PCY7	HNRNPH1	Heterogeneous nuclear ribonucleoprotein H
A0A1X7SBS1	HNRNPU	Heterogeneous nuclear ribonucleoprotein U
P36578	RPL4	60S ribosomal protein L4
A0A0J9YXZ5	IQGAP1	Ras GTPase-activating-like protein IQGAP1
P25705	ATP5F1A	ATP synthase subunit alpha, mitochondrial
P13639	EEF2	Elongation factor 2
A0A087WY00	MYO5A	Unconventional myosin-Va
F8VZ49	HNRNPA1	Heterogeneous nuclear ribonucleoprotein A1
Q5JR95	RPS8	40S ribosomal protein S8
P63244	RACK1	Receptor of activated protein C kinase 1
H7C463	IMMT	MICOS complex subunit MIC60
A0A087WVV2	RRBP1	Ribosome-binding protein 1
Q96C19	EFHD2	EF-hand domain-containing protein D2
V9HW35	HEL-S-55	Peroxiredoxin
C9J0J7	PFN2	Profilin
Q0QEN7	ATP5B	ATP synthase subunit beta
P78527	PRKDC	DNA-dependent protein kinase catalytic subunit
P19474	TRIM21	E3 ubiquitin-protein ligase TRIM21
A0A024R592	GANAB	Glucosidase, alpha neutral AB, isoform CRA_b
Q09666	AHNAK	Neuroblast differentiation-associated protein AHNAK
P62424	RPL7A	60S ribosomal protein L7a
P14649	MYL6B	Myosin light chain 6B
Q5CAQ5	TRA1	Epididymis secretory sperm binding protein
H3BR27	RBMX	RNA-binding motif protein, X chromosome
B1AH77	RAC2	Ras-related C3 botulinum toxin substrate 2
P30050	RPL12	60S ribosomal protein L12
P62277	RPS13	40S ribosomal protein S13
A8MUD9	RPL7	60S ribosomal protein L7
P13010	XRCC5	X-ray repair cross-complementing protein 5
F5H018	RAN	GTP-binding nuclear protein Ran
F8VVM2	SLC25A3	Phosphate carrier protein, mitochondrial
Q5TEU4	NDUFAF5	Arginine-hydroxylase NDUFAF5, mitochondrial
Q6FG43	FLOT2	FLOT2 protein
Q14315	FLNC	Filamin-C
P49411	TUFM	Elongation factor Tu, mitochondrial
P36957	DLST	Dihydrolipoyllysine-residue succinyltransferase component of 2-oxoglutarate dehydrogenase complex, mitochondrial
Q5T985	ITIH2	Inter-alpha-trypsin inhibitor heavy chain H2
P07900	HSP90AA1	Heat shock protein HSP 90-alpha

## Abbreviations

PTC: papillary thyroid carcinoma
DTX: deltex
GEPIA: gene expression profiling interactive analysis
TCGA: the cancer genome atlas
BLCA: bladder urothelial carcinoma
COAD: colon adenocarcinoma
OV: ovarian serous cystadenocarcinoma
PRAD: prostate adenocarcinoma
CESC: cervical squamous cell carcinoma and endocervical adenocarcinoma
READ: rectum adenocarcinoma
STAD: stomach adenocarcinoma
UCEC: uterine corpus endometrial carcinoma
UCS: uterine carcinosarcoma
AJCC: the American Joint Committee on Cancer
FBS: fetal bovine serum
shRNA: short hairpin RNA
cDNA: complementary DNA
qRT-PCR: quantitative real‐time polymerase chain reaction
GAPDH: glyceraldehyde-3-phosphate dehydrogenase
WB: western blot
PMSF: phenylmethanesulfonyl fluoride
SDS-PAGE: sodium dodecyl sulfate-polyacrylamide gel electrophoresis
PVDF: polyvinylidene Fluoride
BSA: bovine serum albumin
IOD: integrated optical density
OD: optical density
MTS: 3-(4,5-dimethylthiazol-2-yl)-5-(3-carboxymethoxyphenyl)-2-(4-sulfophenyl)-2H-tetrazolium
7-AAD: 7-aminoactinomycin D
PI: propidium iodide
RNase A: Ribonuclease A
CO-IP: co-immunoprecipitation
MS: mass spectrometry
WebGestalt: WEB-based GEne SeT AnaLysis Toolkit
GO: gene ontology
KEGG: Kyoto Encyclopedia of Genes and Genomes
STRING: the search tool for the retrieval of interacting genes
PPI: protein-protein interaction
NDUFAF5: NADH Ubiquinone Oxidoreductase Complex Assembly Factor 5
GBM: Glioblastoma
DLBCL: Diffuse large B-cell lymphoma
c-FLIP: cellular FLICE inhibitory protein
EMT: epithelial-mesenchymal transition
NDUFS7: NADH Ubiquinone Oxidoreductase Core Subunit S7
PBS: phosphate-buffered saline
XRCC5: X-ray repair cross-complementing protein 5
